# Generation of a cancer testis antigen mCherry reporter HCT116 colorectal carcinoma cell line

**DOI:** 10.1016/j.heliyon.2018.e00858

**Published:** 2018-10-13

**Authors:** Jyoti B. Chhetri, Elena Drousioti, José Afonso Guerra-Assunção, Javier Herrero, Steen K.T. Ooi

**Affiliations:** a Department of Cancer Biology, UCL Cancer Institute, London, WC1E 6BT, UK; b Bill Lyons Informatics Centre, UCL Cancer Institute, London, WC1E 6BT, UK

**Keywords:** Cell biology, Developmental biology

## Abstract

In the context of cancer immunotherapy, agents that target the immune system to cancer cells need to fulfil two criteria: 1) that they are only expressed on the desired target cell and 2) that they can elicit a potent immunological response. Cancer Testis Antigens are a large disparate family of factors ordinarily expressed in the germ-line but aberrantly expressed across multiple types of cancer. The ability to enforce their expression on tumour cells is an attractive strategy that could render such cells potent targets of the immune system, but very little is known about their regulation. We describe the generation of an mCherry reporter cell line using HCT116 colorectal carcinoma cells that we anticipate will be useful for screen-based approaches to identify novel regulators of CTA expression. Discoveries arising from their use could in future be exploited to enhance tumour cell immunogenicity and improve cancer immuno-therapy.

## 1 Introduction

Harnessing the body's immune system to target and destroy cancer cells holds great promise in the war on cancer and this strategy has recently been shown to be highly effective in clinical trials [1]. Cancer vaccines, a type of immunotherapy, involve exposing the immune system to highly immunogenic, cancer-specific antigens, that subsequently activate both the cellular and humoral branches of the immune system, resulting in the targeted destruction of diseased tissue. Cancer testis antigens (CTA) are a large, disparate family of factors ordinarily expressed in the human germ-line and placenta, with key functions in reproduction and the cell cycle [2].

CTAs were originally identified by autologous typing and serological analysis of cDNA expression libraries (SEREX), which involves the use of a patients' own cytotoxic T-lymphocytes (CTLs) and antibodies respectively, to recognise and respond to antigens produced by their own tumour cells. Originally, 265 unique CTAs classified into 58 different families were identified [3] but a recent study using publically-available data from The Cancer Genome Atlas (TCGA) as well as three separate transcriptomics data sets (The Genotype-Tissue Expression (GTex) Project, Ilumina Human BodyMap 2.0 and Nanjing University Medical University (NJMU) RNA-seq Database) suggests the CTA designation might be applicable to many more factors [4], although it remains unclear whether all of these transcripts can be translated into protein. Typically, CTAs are only expressed in the testis and placenta, and if they are detected in other non-diseased tissues, their expression level is substantially lower than that observed in their native organ.

The testis is an immune-privileged site: the blood-testis barrier combined with the absence of HLA Class I presentation on the surface of germ cells prevents the immune system from interacting and recognising CTAs as self during the establishment of central tolerance [5]. Therefore, from an immunological perspective, CTAs are essentially tumour-specific and hold great promise for cancer vaccines and immunotherapy.

The CTA NUF2 (also known as Cell Division Cycle-Associated Protein 1 CDCA1) is a highly conserved protein originally identified in S. *cerevisiae* and crucial for normal chromosome segregation [6]. As a component of the kinetochore, it is required for the formation of stable microtubule-kinetochore attachments, chromosome alignment and the spindle checkpoint during mitosis in mammalian cells [7]. As well as results from TCGA data, NUF2 overexpression has been reported in a diverse set of cancers including pancreatic [8], gastric and colorectal [9], lung (both small cell and non-small cell), cholangiocellular, urinary bladder and renal [10, 11]. The functional importance of NUF2 in cancer is suggested through screen based studies; an RNAi lethality screen in the Epithelial Ovarian Cancer (EOC) cell line A1847 and other EOC lines identified the factor as important for preventing apoptosis [12]. Analysis of mutations in the PanCancer compendium data set of 4,742 tumours from 21 cancer types revealed mis-sense mutations in NUF2 that could affect chromosome segregation and result in aneuploidy [13]. Proof of the immunogenicity and potential utility of enforcing NUF2 expression for therapeutic purposes comes from a study demonstrating that NUF2 can activate both CD4+/T_h_1 and CTLs [14]. HLA-A24(A*24:02) or HLA-A2(A*02:01)-restricted CTL epitopes were identified that induced tumour-reactive CTLs but not autoimmunity; these epitopes were found to correspond to NUF2 protein. Importantly, long peptides were identified that were capable of activating both T_h_1 and CTLs.

Despite their general expression across multiple types of cancers, an outstanding issue is that CTA expression is heterogeneous i.e. not all cases of a particular cancer present with CTA expression, and even within tumors that express or upregulate CTA expression, expression does not occur uniformly within all cells [15]. Epigenetic mechanisms, particularly 5-methyl cytosine (5mC) found in CG contexts (DNA methylation), appear to be involved in CTA silencing. The general DNA demethylating drug, 5′-aza-2′deoxycytidine (5-AZA-CdR) activates MAGE-A1 expression in human melanoma cells [16]. Studies in mouse indicate that promoter methylation arises during preimplantation development [17] and recent studies indicate the importance of transcription factor binding in driving the loss of DNA methylation marks around promoter regions [18] whilst its presence can antagonise transcription factor binding [19]. These observations are consistent with a model whereby transcription factors play a dominant role in activating CTA expression, followed by the loss of DNA methylation that may assist in maintaining gene activation.

Forward based screens, for example using the CRISPR/Cas9 system and single guide RNAs (sgRNAs), provide a powerful means of identifying novel factors functionally involved in biological processes [20]. How CTAs are correctly regulated remains poorly defined and improvements in our understanding of the basic mechanisms involved will be crucial in the future development of targeted cell therapies designed to activate their expression in non-expressing cells. Using CRISPR/Cas gene editing, we describe the generation of a NUF2-mCherry reporter in HCT116 colorectal carcinoma cells. We propose that these cells will be of utility in screen-based approaches to identify novel regulators of CTA expression.

## 2 Materials and methods

### 2.1 NUF2 expression analysis of TCGA and GTex data

Expression data for different cancer types analysed was downloaded from The Cancer Genome Atlas (TCGA) Research Network (http://cancergenome.nih.gov/). A custom Perl script extracted expression scores for NUF2 and generated the median for each type of cancer. For tissue expression data, tissue-filtered box plots were downloaded from the GTex Portal (https://www.gtexportal.org/home/).

### 2.2 Generation of *NUF2*^*mCherry*^ allele by CRISPR/Cas gene editing

A suitable sgRNA targeting the *NUF2* locus close to the STOP codon was designed using the CHOPCHOP web tool (http://chopchop.cbu.uib.no/index.php) [21, 22]. A replacement cassette containing the mCherry coding DNA sequence (CDS) lacking both a start ATG and stop codon, flanked by 500bp homology arms either side of the Cas9 cut site was generated by Gibson Assembly of gene blocks (IDT) into pJET1.2 cloning vector (Thermo Fisher Scientific). To prevent hCas9-mediated restriction following incorporation of the *mCherry* CDS by Homology Dependent Repair (HDR), a silent mutation was incorporated into the replacement cassette that eliminated the Protospacer Adjacent Motif (PAM) sequence, changing the sequence from AGG to AGA, which retained coding for arginine. Following sequencing to confirm correct assembly into pJET1.2, the replacement cassette was liberated by restriction digestion of *BamHI* sites engineered at the ends of the homology arms and the DNA was used for transfection of HCT116 colorectal carcinoma cells [23].

### 2.3 HCT116 cell culture

Cells were cultured in HCT116 cell media which consisted of Dulbecco modified Eagle medium (DMEM) supplemented with 100 IU/ml penicillin and 10% fetal bovine serum. Cell counting was performed using Muse^TM^ Count & Viability Kit (Millipore) on a Muse^TM^ Cell Analyzer (Millipore).

### 2.4 qRT-PCR analysis of NUF2 expression

For HCT116 cells, RNA was extracted using Trizol reagent (Thermo Fisher Scientific). For human lung, RNA was prepared using AllPrep RNA/Protein Kit (Qiagen). Human Testis total RNA was purchased from Clonetech. Except for lung RNA, RNAs were additionally treated with Turbo DNase (Thermo Fisher Scientific). First strand cDNA was generated using Maxima Reverse Transcriptase (Thermo Fisher Scientific) and oligo(dT)_18_ primers according to the manufacturer's protocol. cDNAs were diluted 1:10 and used for qRT-PCR using the Roche Universal Probe Library System (Roche). Primers compatible with hydrolysis Probe 8 were used and GAPDH probe and primer set used as the reference gene control. Reactions were run on a LightCycler® 480 II (Roche).

### 2.5 Transfection of HCT116 cells

The day before transfection, 2.5 × 10^5^ cells were seeded onto the well of a 24 well plate. On the day of transfection, cells were transfected using Fugene 6 transfection reagent (Promega) according to the manufacturer's protocol. Per transfection, a total of 1 μg of DNA was used. This consisted of hCas9 plasmid (400 ng) [24], a synthetic gRNA expression cassette containing NUF2 gRNA (300 ng), an *mCherry* CDS-containing replacement cassette (200 ng) and a synthetic gRNA expression cassette containing *HRPT* gRNA (100 ng). The latter was included to enrich for transfected cells by culture in 6-Thioguanine (6-TG) containing media. Synthetic gRNA expression cassettes were designed as described by Mali and colleagues [24]. 16 to 18 hours post transfection, cells were harvested, counted and seeded onto four 15 cm plates (2 × 10^5^ cells/plate). Three days after seeding, media was replaced with 6-TG containing media (final concentration, 10 μg/ml). Fresh 6-TG containing media was replaced every four days. Twelve days after initial transfection, individual colonies were identified by eye, picked and cultured in flat-bottom 96-well plates. Cells were expanded for DNA preparation and continued culture.

### 2.6 PCR screening and sequencing

Genomic DNA from confluent 96 well plates was prepared using Quick-DNA^TM^ 96 Kit (Zymo Research) according to the manufacturer's protocol. PCR was performed using KAPA2G Robust HotStart PCR Kit (Kapa Biosystems) according to the manufacturer's protocol with the inclusion of Enhancer reagent. For the generation of amplicons for Sanger sequencing, PCR was optimised using Phusion High-Fidelity DNA Polymerase (Thermo Fisher Scientific) and gel resolved amplicons purified using MinElute Gel Extraction Kit (Qiagen) according to the manufacturer's protocol.

### 2.7 Sequences

For screening/genotyping PCRs, the following primers were used (listed 5′ to 3′): NUF2_5′_F1 (agcacatccatataggcttgaa), NUF2_WT_R1 (gccagttgcatcaatgaaga), NUF2_5′_R1 (acatgaactgaggggacagg), NUF2_3′_F1 (gaccacctacaaggccaaga), NUF2_3′_R1 (tacctggagcttccatgacc). The sgRNA sequences used for targeting hCas9 were: NUF2_gRNA14 (CGGTATTGAAAAGGCAGCAGAGG), HPRT_gRNA (aaagtaattcacttacagtctgg). For NUF2 qRT-PCR, the primers used were: NUF2_8_F (ccagacaagaagtggtggaga) and NUF2_8_R (tgatataactgcacttccaactgac).

### 2.8 Antibodies

The following antibodies were used at the dilutions indicated: Anti-Tubulin (1:10,000, T6199, Sigma-Aldrich), Anti-RFP (1:700, 6G9, Chromotek) and Anti-NUF2 (1:100, sc-271-251, Santa Cruz).

### 2.9 Western blot

Pelleted cells (fresh or snap-frozen) were lysed in RIPA buffer (1 × PBS, 1 % NP40, 0.5% Sodium Doxycholate, 0.1% SDS). Samples were quantified using Bio-Rad Protein Assay (Bio-Rad) and equal amounts (50 μg) of lysate were loaded onto 8% SDS-PAGE gels. Following separation by electrophoresis, proteins were transferred onto Nitrocellulose membrane (GE Healthcare), blocked in PBST (1 × PBS + 0.1% Tween-20) + 5% milk for one hour before incubating with primary antibodies for two hours. Membranes were washed in PBST, incubated with secondary antibody, washed in PBST, before HRP signal development using Luminata Forte Western HRP Substrate (Millipore). For anti-NUF2 antibody, TBS was used instead of PBS. Signals were detected by exposure of membranes onto Hyperfilm ECL (Amersham).

### 2.10 FACS analysis

Cultured HCT116 and *NUF2*^*mCherry/mCherry*^ cells were harvested in 0.25% trypsin and re-suspended in 1 × Dulbecco's PBS. Two volumes of 100% ice-cold ethanol were added for fixation and cells were incubated overnight at −20 ^°^C. The next day, cells were washed in 1 × PBS and re-suspended in FACS buffer (1 × PBS, 1 × BSA and 10 μg/ml RNase A). Cells were incubated on ice for 15 minutes with 5 mM DRAQ5^TM^ Fluorescent Probe Solution (Thermo Fisher Scientific) before acquisition on a BD LSRFortessa X-20 (BD Biosciences) flow cytometer. Analysis was performed using FlowJo software (FlowJo).

### 2.11 Cell cycle and viability analysis

Cultured HCT116 and *NUF2*^*mCherry/mCherry*^ cells were harvested and labelled using Muse^TM^ Cell Cycle and Annexin V & Dead Cell Kit (Millipore) according to the manufacturer's protocol before running on the corresponding setting on a Muse^TM^ Cell Analyzer (Millipore).

### 2.12 Cell imaging

Images of live cells in culture were acquired on a Zeiss Axio Observer Z1 (Carl Zeiss) microscope. For confocal imaging, cells were harvested, fixed in 2% PFA and seeded by centrifugation (800 rpm, 4 minutes) onto coated slides using a Shandon Cytospin (Thermo Fisher Scientific). After briefly washing with 1 × PBS, VECTASHIELD Antifade Mounting Medium with DAPI (Vectorlabs) was applied and slides covered with cover slips. Images were acquired on an LSM 880 with Aryscan microscope (Zeiss).

## 3 Results and discussion

**Fig. 1:**
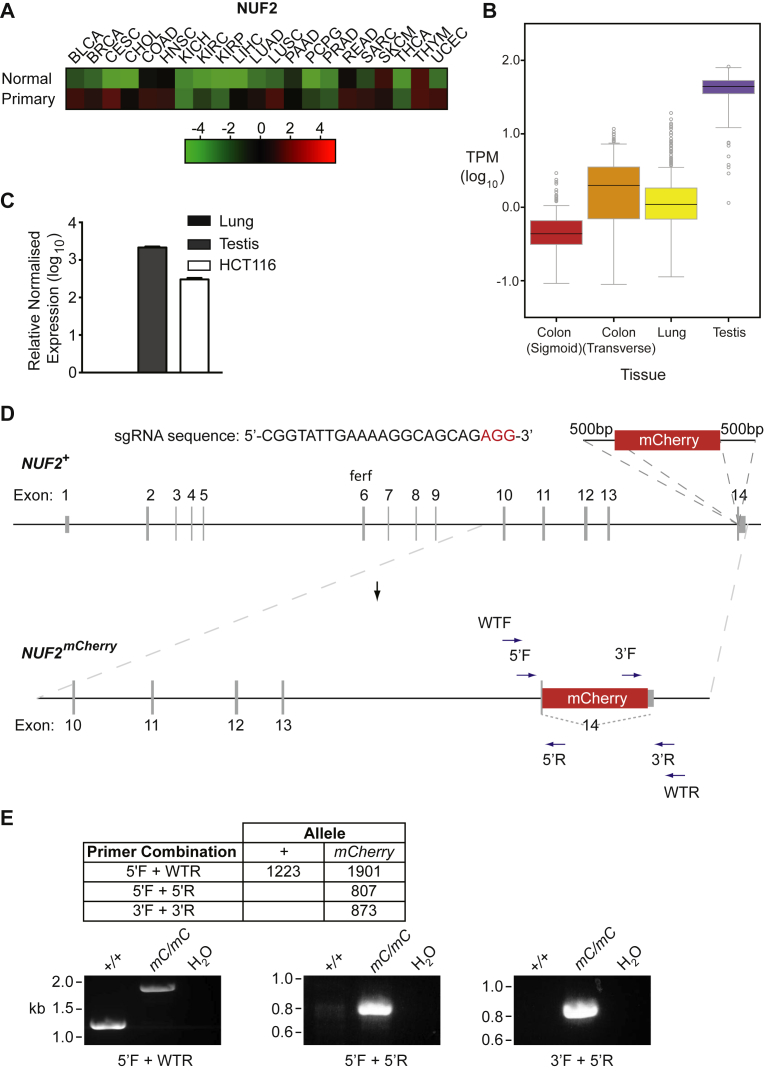
Generation of *NUF2*^*mCherry/mCherry*^ reporter cell line in HCT116 cells. (A) Heat map of median NUF2 expression scores in normal and primary tumour tissues in the following different cancers: breast invasive carcinoma (BRCA); bladder urothelial carcinoma (BLCA); cholangiocarcinoma (CHOL); colon adenocarcinoma (COAD); head & neck squamous cell carcinoma (HNSC); kidney chromophobe (KICH); kidney renal clear cell carcinoma (KIRC); kidney renal papillary cell carcinoma (KIRP); liver hepatocellular carcinoma (LIHC); lung adenocarcinoma (LUAD); lung squamous cell carcinoma (LUSC); mesothelioma (MESO); prostate adenocarcinoma (PRAD); rectum adenocarcinoma (READ); thyroid carcinoma (THCA); uterine corpus endometrioid carcinoma (UCEC); (B). Box plots showing RNA-seq data of *NUF2* expression in different tissues indicated. Log_10_ expression level measured as Transcripts Per Million (TPM). Lines indicate median and 25^th^ and 75^th^ percentiles. Values shown for each tissue are median and sample size, respectively: Colon (sigmoid) (0.386, 233); Colon (transverse) (1.933, 274); Lung (1.045, 427) and Testis (44.18, 259). Data obtained from the GTex Portal (https://www.gtexportal.org/home/); (C) Bar graphs showing relative, GAPDH-normalised qRT-PCR expression levels of *NUF2* in HCT116 cells. Log_10_ expression levels in human lung and testis are shown as controls (negative and positive, respectively). Expression is shown relative to levels in lung; (D) Diagram showing CRISPR/Cas gene editing strategy used. Sequence of sgRNA used to target hCas9 to the *NUF2* locus is shown. Primers used in targeting the *NUF2* locus are located outside the homology arms; (E) PCR results of targeted *NUF2*^*mCherry/mCherry*^ clones (labelled *mC/mC*). Table shows expected amplicon sizes using primers indicated in D. Images of gel resolved amplicons using the primer combinations indicated stained with ethidium bromide and visualised under UV light.

Analysis of The Cancer Genome Atlas (TCGA) data revealed that the CTA NUF2 is consistently over-expressed across a broad range of different cancer types (Fig. 1A). Analysis of publicly available RNA-Seq data through the GTex Portal indicates that normal colon has low levels of NUF2 expression, similar to that observed in normal lung (Fig. 1B). qRT-PCR confirmed that whilst NUF2 expression is undetectable in non-germ line tissue (in this case lung), it is 2-3 orders of magnitude higher in both testis and the colorectal carcinoma cell line, HCT116 (Fig. 1C). To generate a reporter cell line as a tool for screen-based approaches to identify novel regulators of NUF2 expression, we used CRISPR/Cas editing to introduce an *mCherry* CDS into the endogenous *NUF2* locus of HCT116 colorectal carcinoma cells to generate an in-frame fusion protein. As this gene has a promoter-associated CpG island that may be involved in regulating its expression, the *mCherry* CDS was added to the C-terminus, immediately before the endogenous STOP codon in exon 14 (Fig. 1D). CHOPCHOP web tool was used to identify a suitable sgRNA sequence, close to the STOP codon. To enrich for successfully targeted cells, an sgRNA targeting the *HPRT* locus which has previously been used in HCT116 cells was also included [25]. Two separate transfections were performed and a total of 576 colonies were picked and screened by PCR, initially using primers to detect the *mCherry* CDS. 13 clones were found to carry the *mCherry* reporter and were expanded up for further analysis. Using the primers indicated in Fig. 1C and D, which are external to the regions used for the homology arms, PCR on DNA extracted from one clone (P1_E2) generated amplicons at the expected sizes following successful targeting (Fig. 1E and Supplemental Fig. S1). PCR using primers 5′F + WTR, designed to detect the wildtype untargeted allele failed to generate an amplicon at the expected size indicating these cells are homozygous for insertion of the *mCherry* reporter; these cells are hereafter referred to as *NUF2*^*mCherry/mCherry*^*(mC/mC)*.

**Fig. 2:**
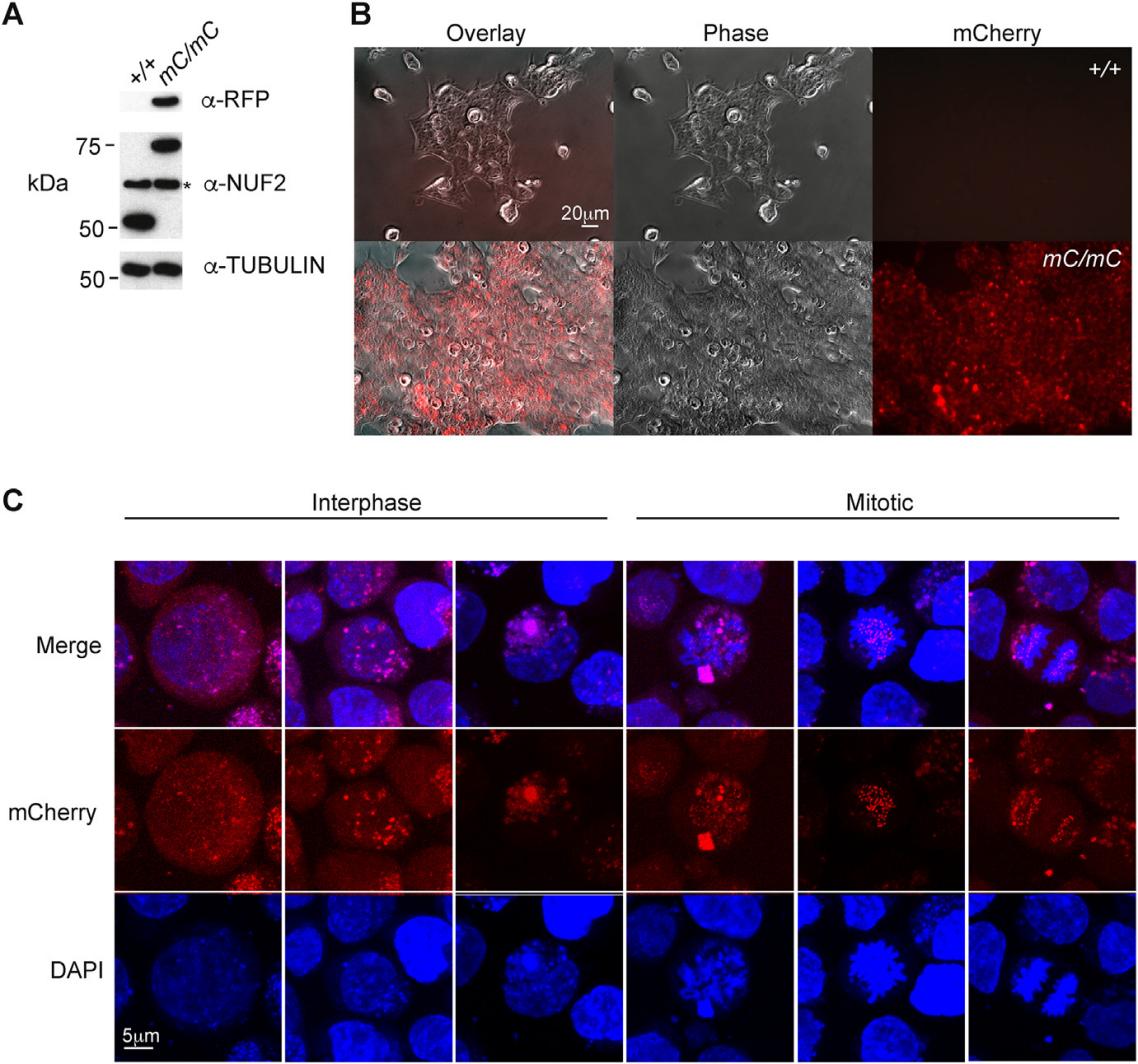
Characterisation of NUF2-mCherry expression. (A) Western blot using antibodies indicated on RIPA lysates from parental untargeted HCT116 cells as well as *NUF2*^*mCherry/mCherry*^ cells. For anti-NUF2 antibody, a non-specific band was observed, as indicated by *. Anti-Tubulin antibody signal used as a loading control; (B) Micrograph images showing fluorescence signal from live HCT116 and *NUF2*^*mCherry/mCherry*^ cells; (C) Confocal micrograph images showing fluorescence signals in *NUF2*^*mCherry/mCherry*^ cells. Nuclei were revealed using DAPI stain and used to distinguish interphase from mitotic cells.

The strategy of using an *mCherry* CDS lacking various elements necessary for successful transcription (i.e. promoter, start ATG, stop codon, polyadenylation signal) was to ensure that mCherry expression would only be detected following correct insertion into the *NUF2* locus in the manner anticipated. Expression of NUF2-mCherry fusion protein was confirmed using several approaches. Western blot analysis was performed on whole cell extracts/RIPA lysates using both anti-RFP and anti-NUF2 antibodies. NUF2 protein is predicted to be approximately 54 kDa; the inclusion of mCherry generates an approximately 80kDa protein and an immunoblot signal was observed at this size in *mC/mC* cells (Fig. 2A and Supplemental Fig. S2). This result was recapitulated when lysates were probed with anti-NUF2 antibody. In addition to the fact that the size of the band is increased in *mC/mC* cells, we note the presence of a non-specific band approximately 60kDa in size (Fig. 2A, *) present in both the untargeted parental HCT116 and *mC/mC* cells. Analysis of live cells by fluorescence microscopy indicated robust mCherry expression (Fig. 2B). NUF2 is known to have a major function during metaphase to ensure microtubules correctly attach to kinetochores. We further examined the subcellular localisation of NUF2-mCherry protein by confocal microscopy (Fig. 2C). In interphase cells, NUF2-mCherry displayed a more diffuse distribution although discrete signals were also observed. The cytoplasmic nature of the protein agrees with previous observations in both human A341 epidermoid carcinoma and HEK 293 cells (https://www.proteinatlas.org/ENSG00000143228-NUF2/cell#human). In mitotic cells, NUF2-mCherry distribution became more punctate and co-localised with chromosomal regions most likely to be centromeres, at stages indicative of metaphase and anaphase. These results agree with earlier studies examining the sub-cellular distribution of GFP-tagged NUF2 protein in DT40 cells [26], and suggest that NUF2-mcherry protein is capable of correct localisation during the cell cycle.

**Fig. 3:**
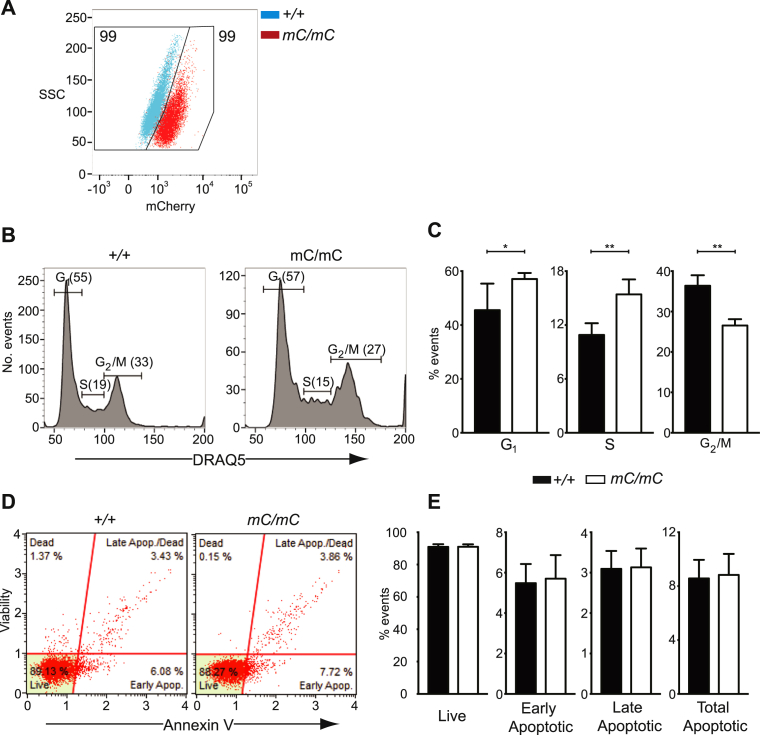
Characterisation of NUF2-mCherry cell line proliferation and viability. (A) FACS scatter plot of HCT116 cells overlaid with *NUF2*^*mCherry/mCherry*^ cells. Numbers are percentages of live and single-gated events within gates indicated; (B) Histograms showing DRAQ5^TM^ staining profile of cell lines indicated. Populations divided into G_1_, S and G_2_/M. Numbers indicate percentage of live-gated cells; (C) Bar graphs showing percentage of cells at different cell cycle stages indicated. Exact p-values (n = 6, Mann-Whitney) are 0.0152 (G0/G1), 0.0043 (S) and 0.0022 (G2/M); (D) FACS plots of quadrant-gated HCT116 cells labelled with Muse^TM^ Annexin & Dead Cell Assay reagent for viability analysis. Quadrants indicated are live, early apoptotic, late apoptotic and dead; (E) Bar graphs showing percentage of cells in the different viability groups indicated. Exact p-values (n = 6, Mann-Whitney) are 0.9372 (live), 0.9372 (early apoptotic), 0.9372 (late apoptotic) and >0.999 (total apoptotic).

We next assessed the ability to detect NUF2-mCherry signal by FACS. The mCherry expression profile for *mC/mC* cells, was shifted relative to signal observed in parental, untargeted HCT116 cells (Fig. 3A). Cell cycle analysis of live-gated cells using a fluorescent DNA dye (DRAQ5^TM^) revealed that mCherry is expressed throughout the cell cycle (Fig. 3B), consistent with our immunofluorescence data. Quantitative analysis of the percentage of cells at different stages of the cell cycle revealed a significant increase in cells in both G1 and S phase with a concomitant reduction in the proportion in G2/M phase (Fig. 3C). Combined with our microscopy data, this suggests that although NUF2-mCherry correctly localises during mitosis, the presence of the mCherry moiety may be affecting NUF2 function. The human genome reportedly encodes twenty cyclin dependent kinases (CDKs) and twenty-nine cyclins (CLNs) [27]. While we cannot formally exclude any direct effect on the levels of the various cyclin proteins expressed throughout the cell cycle, correct NUF2 function is known to be required for the formation of stable microtuble-kinetochore attachments and subsequent passage through the mitotic spindle checkpoint. As these processes are regulated by Cyclin A proteins [28], we consider these to be candidate cyclins whose expression is perturbed by the presence of the mCherry moiety on NUF2 protein. Despite this effect on the cell cycle, this did not translate to any significant effect on cell viability (Figs. 3D and E).

The successful exploitation of CTAs for use in cancer vaccines requires improvements in our understanding of how these factors are regulated. Mammalian gametogenesis, particularly spermatogenesis is a highly controlled process and there is likely to be co-ordinate regulation of these factors. Presently, little is known about how *NUF2* expression is regulated at the transcriptional level. A single, previous study in HCT116 cells claimed that the highly abundant protein heterogeneous nuclear ribonucleoprotein K (HNRNPK) is an important transcriptional activator of *NUF2* [29]. Despite a number of attempts, we have been unable to generate *HNRNPK*-deficient cells in order to both validate the role of HNRNPK in regulating NUF2 expression as well as to serve as a positive control in order to test our cell line. However, we anticipate that the cell line described in this present study will serve as a useful tool in future screen-based approaches designed to identify novel regulators of NUF2 expression.

We note that these labelled cells could also be suitable for tracking purposes in pre-clinical mouse models of colorectal cancer. HCT116 and other colorectal cancer cell lines have been successfully used in a number of transplantation models involving immunodeficient mice, using both subcutaneous and orthotopic xenografts [30]. Labelling studies have also been performed involving the transduction of colorectal cancer cell lines with fluorescent reporter transgenes and injecting/implanting polyclonal populations of such cells [31, 32], which are likely to have variable reporter expression. The fact that the reporter cell line we have generated carries the mCherry reporter fused to an endogenous gene, controlled by its endogenous regulatory elements indicates more physiological and more importantly, stable regulation of reporter expression. This would be an important and useful feature for longitudinal studies. As mCherry fluorescence from these cells is readily detectable by FACs, these cells would be useful in monitoring and quantifying the spread of tumour growth both within organs and possibly by metastasis. They could therefore serve as a tool for preclinical studies testing the effectiveness of different therapeutic strategies. Finally, the presence of the mCherry moiety fused to NUF2 could be utilised for both live cell imaging and protein purification studies designed to further investigate mammalian mitosis.

## Declarations

### Author contribution statement

Jyoti B. Chhetri, Elena Drousioti: Performed the experiments.

Jose Afonso Guerra-Assuncao, Javier Herrero: Contributed reagents, materials, analysis tools or data.

Steen K.T. Ooi: Conceived and designed the experiments; Performed the experiments; Analyzed and interpreted the data; Wrote the paper.

### Funding statement

This work was supported by an ERC Starting Grant (281784-XXDNAM) and by the CRUK-UCL Centre Development Fund Award (C416/A18088).

### Competing interest statement

The authors declare no conflict of interest.

### Additional information

No additional information is available for this paper.
