# Supplementary figures and images for: Generation of a cancer testis antigen mCherry reporter HCT116 colorectal carcinoma cell line
Source: Heliyon. 2018 Oct 13;4(10):e00858. doi: 10.1016/j.heliyon.2018.e00858 (PMC6197641; doi:10.1016/j.heliyon.2018.e00858)

Supplemental Figure S1

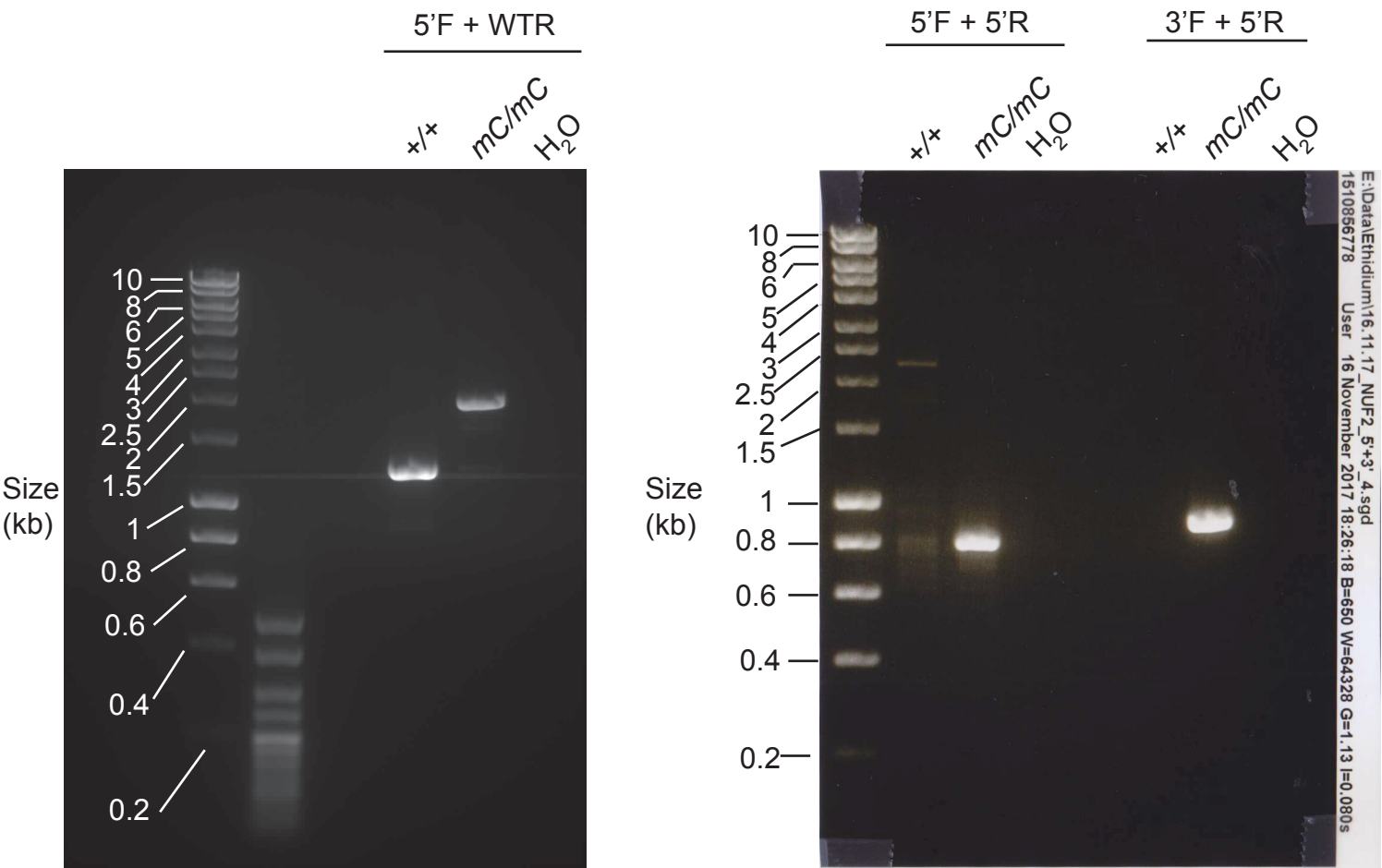

Supplement: Supplemental Figure S1 [file mmc1.pdf]

Supplemental Figure S2

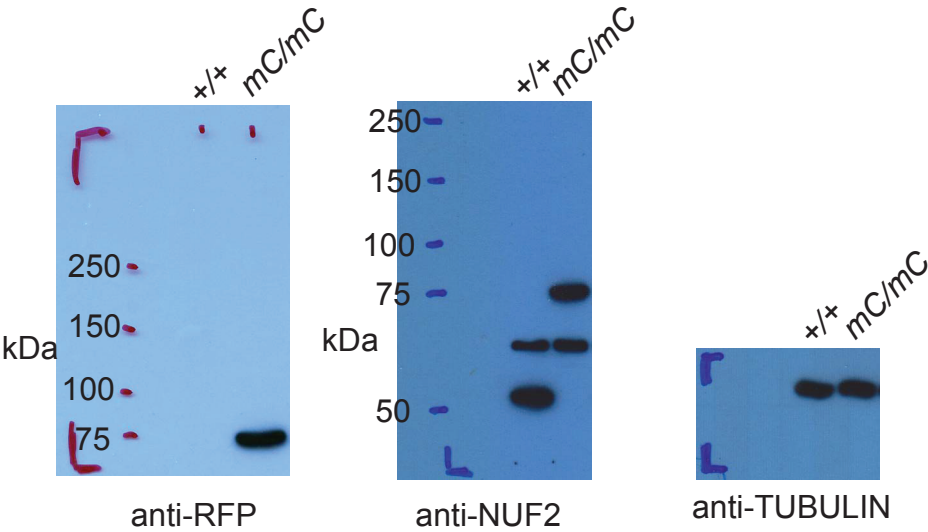

Supplement: Supplemental Figure S2 [file mmc2.pdf]
