# Supplementary material for: Generation of a cancer testis antigen mCherry reporter HCT116 colorectal carcinoma cell line
Source: Heliyon. 2018 Oct 13;4(10):e00858. doi: 10.1016/j.heliyon.2018.e00858 (PMC6197641; doi:10.1016/j.heliyon.2018.e00858)
Supplement: Supplemental Material Figure Legends [file mmc3.docx]

**­Supplementary Material Information**

**Generation of a Cancer Testis Antigen (CTA) mCherry reporter HCT116 colorectal carcinoma cell line**

Jyoti B. Chhetri, Elena Drousioti, José Afonso Guerra-Assunção, Javier Herrero & Steen K.T. Ooi

**Supplemental Figure S1.** Uncropped images for all UV-visualised ethidium stained gels displayed in Figure 1E.

**Supplemental Figure S2.** Uncropped images for all western blot images displayed in Figure 2A.
